# Multivector model predictive control for multiphase induction machines with dead-time knowledge

**DOI:** 10.1038/s41598-026-46936-6

**Published:** 2026-04-07

**Authors:** Juan Carrillo-Rios, Manuel Cordoba-Ramos, Rafael Lara-Lopez, Ignacio Gonzalez-Prieto, Mario J. Duran, Juan Jose Aciego, Pablo Mora-Moreno, Angel Gonzalez-Prieto

**Affiliations:** 1 Department of Electrical Engineering, University of Malaga, Malaga, 29010 Spain; 2 Department of Electrical Engineering, Polytechnic University of Madrid, Madrid, 28040 Spain

**Keywords:** Energy science and technology, Engineering, Mathematics and computing

## Abstract

As requirements in the field of energy efficiency and transport have become more demanding, multiphase technology has begun to emerge in commercial applications in order to meet these demands. In terms of multiphase electric drive regulation, model predictive control has gained traction thanks to its fast dynamic response, flexibility and easy control design. Furthermore, the development of implicit modulators such as multivector solutions has provided these schemes with competitive current quality results. However, the use of higher sampling frequencies in the pursuit of an improved performance presents a blind spot for these control solutions. More concretely, the dead time of voltage source converters (VSCs) has an increasing impact on multiphase electric drives as the sampling frequency becomes higher. The present work first analyses how this phenomenon affects the uniquely available subspaces which characterize multiphase systems. Following these results, dead-time information is added in a multivector predictive scheme and the cost function is reformulated to mitigate its impact. Different operating metrics have been employed to illustrate the effectiveness of the proposed algorithm to increase current quality in a six-phase drive when dead-time effect appears.

## Introduction

Multiphase machines have become an interesting solution for industry applications where high power and reliability are required^1–3^. By presenting more than three phases, these devices bring forth numerous advantages such as inherent fault tolerance, better power density and higher efficiency^4^. However, the exploitation of these desirable traits was limited by the control complexity of these machines, which needed to account for the extra degrees of freedom that emerged from the increased number of phases^5^. Fortunately, the advances in the field of digital signal processing have procured a renaissance of multiphase systems both in academia and industry, with companies implementing multiphase motors in different electric vehicle systems^6–8^.

Focusing on control algorithms for multiphase electric drives, finite control set model predictive control (FCS-MPC) has appeared as an appealing option in the last decade^9^. Its main benefits include high flexibility thanks to the existence of a cost function^10–15^ and fast dynamic response due to a notable use of the dc-link voltage^16^. Nevertheless, the higher number of phases produces the appearance of secondary subspaces whose currents must be carefully controlled due to their impact on harmonic injection^5^. In order to tackle this problem, one suitable approach is the use of multivector solutions, which are based on applying more than one voltage vector per control cycle^17–19^. When these vectors are selected with the intention of ideally producing null average voltages in the secondary subspaces, the current harmonic injection can be greatly reduced, leading to an improved performance. Through this method, FCS-MPC has achieved a lower harmonic distortion than field-oriented control (FOC), without the need for an explicit modulator^5^.

Even though multivector FCS-MPC based on virtual voltage vectors (VVs) has successfully regulated multiphase machines^11^, it also presents limitations. Some well-known disadvantages are the worse utilization of the dc-bus voltage, which leads to lower achievable modulation indices and less flexibility when VVs are calculated offline^11^. However, an often overlooked limitation in multiphase FCS-MPC with VVs is the higher sensitivity to dead-time effects^20^. Since the multivector approach applies more than one control action per cycle, the time of application of each switching state is lower for the same sampling period^21^. Consequently, the proportion of the dead time in the voltage source converter (VSC) with respect to the application time is higher. In this situation, transitional voltage vectors caused by VSC switches can become a critical issue^22^. The mitigation of this phenomenon in multiphase drives can be essential, since their secondary subspaces typically present low impedance values, resulting in high current disturbances that can jeopardize control quality^5,11^.

Unfortunately, there is another factor that negatively contributes to the dead-time problem, the decrease in sampling period. Short control times are desirable to obtain fast regulation responses and overall improved performance. Nevertheless, despite these implementation conditions being ideal for improving the system behavior, the impact of dead time notably increases since the specific duty cycle of each switching state of the applied VVs decreases. This undesirable effect was observed in^20^, where the increase of the sampling frequency caused a higher injection of low-order harmonic currents. Therefore, in order to exploit the desirable traits of multivector FCS-MPC strategies and higher control frequencies, it becomes mandatory to consider the dead-time influence.

Due to the importance of the dead-time phenomenon, many studies have been performed, each proposing a suitable answer according to their control scheme and machine configuration. For regulation methods with an explicit modulator, one popular solution is to modify the reference voltage values to compensate for the mismatch between the ideal voltage output and the real one caused by the dead time^23,24^. For the same purpose, the application of dual resonant controllers targeting the fifth and seventh harmonics in the secondary plane has been successfully demonstrated in^25^. However, this approach is not suitable for discrete FCS-MPC, since it does not select the control action based on a voltage reference. Focusing on previous solutions applicable to multiphase direct schemes, a dead-time compensation method is suggested in^26^ for an open-end winding (OeW) nine-phase machine where VVs based on three switching states are used as control actions. This proposal injects a fourth voltage vector that is aligned with the VV in the $\alpha$-$\beta$ plane and in quasi-opposite direction of the dead-time voltage vectors in the remaining four *x*-*y* subspaces. A follow-up proposal is presented in^27^ where, instead of applying a fourth vector to achieve dead-time compensation, a second set of VVs are defined and applied alongside the original VVs every control cycle with this objective. These methods successfully compensate the dead-time effect at the expense of a higher complexity and switching losses, but the solution is specific for OeW configuration where a huge amount of switching states ($>10^4$) are available for the cancellation procedure.

As an alternative, the dead-time impact on the current quality can be reduced by informing the control scheme of its existence, as it has been observed in three-phase systems^22^. By accounting for the voltage modification, the FCS-MPC algorithm can improve its decisions and performance. Extending this approach to multiphase electric drives for the first time, this work firstly studies how the VSC dead time affects both the primary and secondary subspaces. This initial contribution provides further insight into the problem of using VV-based FCS-MPC at increasingly higher switching frequencies. After this analysis, a second contribution includes the implementation of an improved multivector FCS-MPC equipped with knowledge of dead-time effect and backed by experimental results. This proposal for multivector FCS-MPC strategies opens the possibility to keep on employing VVs with a satisfactory performance in a higher range of switching frequencies. The major advantages of the proposed FCS-MPC approach compared to the current state of the art are as follows:

- It is implementable in FCS-MPC algorithms^23–25^.
- It avoids the need to add additional switching states to the VVs, thereby preserving the number of commutations in each control period^26,27^.
- It minimizes the instantaneous contribution in the secondary subspace due to the selected active control actions^28^.
- It eliminates the need to implement specific controllers for each harmonic order^25^.
- It is specifically designed for multiphase drives^22^.
- It avoids the need to reorder switching states during the control period^28^.

The manuscript is structured as follows. First the topology of a six-phase system based on an induction machine (IM) is reviewed. The next section details how to estimate the influence of the dead time in the subspaces that model the machine’s performance. Later FCS-MPC for both single and multivector schemes is detailed, further illustrating the importance of the dead-time phenomenon when a higher switching frequency is reached. Next, the proposed multivector method and experimental comparative results are presented. Finally, the conclusions of the work are detailed.

## Six-phase induction motor drive

**Fig. 1:**
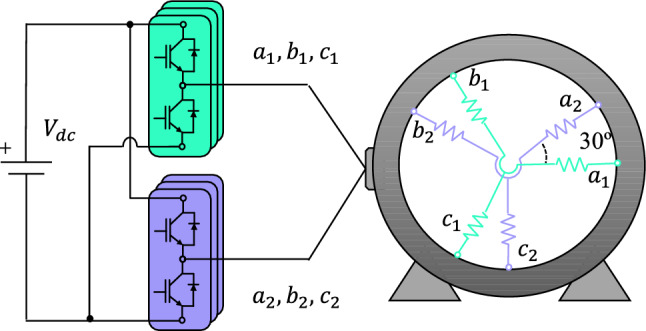
Topology of the asymmetrical six-phase IM with a double three-phase two-level VSC.

The selected electrical drive presents two main components: a six-phase IM and a dual three-phase two-level VSC (see Fig. 1). The stator of the electrical IM is formed by two three-phase windings shifted 30$^\circ$ and configured with two isolated neutral points. The VSC is connected to a single dc link, providing $2^6=64$ switching states. The VSC behavior can be expressed using a vector [*S*] where its $S_{ij}$ components illustrate the state of each VSC leg. Subscripts $i \in \{a,b,c\}$ and $j \in \{1,2\}$ indicate phase and stator winding, respectively. $S_{ij}$ equals 1 if the upper switch of the leg is ON and the lower switch is OFF, whereas $S_{ij}$ would equal 0 if the opposite scenario occurs. The values of the stator phase voltages ($v_{ij}$) can be estimated using [*S*] and the dc-link voltage ($V_{dc}$) :

1 $$\begin{aligned} \begin{bmatrix} \begin{array}{c} v_{a1} \\ v_{b1} \\ v_{c1} \\ v_{a2} \\ v_{b2} \\ v_{c2} \end{array} \end{bmatrix} = \frac{V_{dc}}{3} \begin{bmatrix} \begin{array}{rrrrrr} 2 & -1 & -1 & 0 & 0 & 0 \\ -1 & 2 & -1 & 0 & 0 & 0 \\ -1 & -1 & 2 & 0 & 0 & 0 \\ 0 & 0 & 0 & 2 & -1 & -1 \\ 0 & 0 & 0 & -1 & 2 & -1 \\ 0 & 0 & 0 & -1 & -1 & 2 \end{array} \end{bmatrix} \begin{bmatrix} \begin{array}{c} S_{a1} \\ S_{b1} \\ S_{c1} \\ S_{a2} \\ S_{b2} \\ S_{c2} \end{array} \end{bmatrix} . \end{aligned}$$

Phase variables can be used to describe the behavior of the drive. However, some reference frames have been widely employed to simplify understanding and control. In this regard, vector space decomposition (VSD) is the most popular solution^29^. Applying VSD approach, phase variables can be transformed onto three orthonormal subspaces with specific physical meaning. The first subspace is $\alpha$-$\beta$, which is related to flux and torque production, whereas the second plane (*x*-*y*) only represents stator copper losses when the machine has distributed windings and spatial harmonics can be neglected. Finally, it is relevant to highlight that zero-sequence has no influence on control strategies since the isolated neutral configuration does not allow these currents to flow.

The Clarke transformation matrix (2) based on the stator winding configuration^30^ permits expressing phase variables onto VSD components:

2 $$\begin{aligned} & \begin{aligned} \mathbf {[C]}&= \frac{1}{3} \begin{bmatrix} 1 & -1/2 & -1/2 & \sqrt{3}/2 & -\sqrt{3}/2 & 0 \\ 0 & \sqrt{3}/2 & -\sqrt{3}/2 & 1/2 & 1/2 & -1 \\ 1 & -1/2 & -1/2 & -\sqrt{3}/2 & \sqrt{3}/2 & 0 \\ 0 & -\sqrt{3}/2 & \sqrt{3}/2 & 1/2 & 1/2 & -1 \\ 1 & 1 & 1 & 0 & 0 & 0 \\ 0 & 0 & 0 & 1 & 1 & 1 \end{bmatrix} \end{aligned} , \end{aligned}$$

3 $$\begin{aligned} & \begin{bmatrix} v_{\alpha s} ,\! v_{\beta s} ,\! v_{xs},\! v_{ys}, \!v_{z1},\! v_{z2} \end{bmatrix} \!^\text {T} \!\!\!=\!\! \mathbf {[C]}\!\! \cdot \!\! \begin{bmatrix} \! v_{a1}, \!v_{b1},\! v_{c1}, \!v_{a2}, \!v_{b2}, \!v_{c2} \end{bmatrix}^\text {T}\!\!\!\!. \end{aligned}$$

**Fig. 2:**
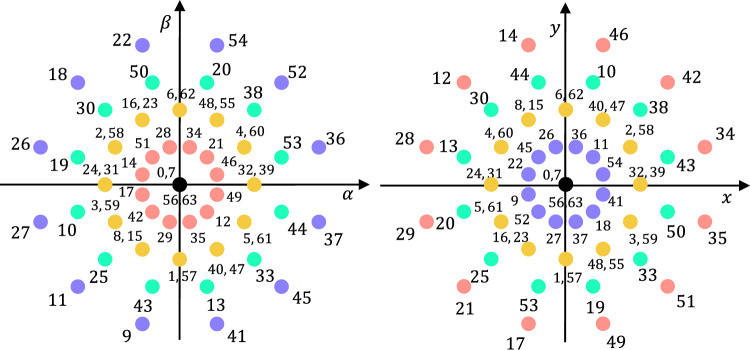
Voltage vector distribution in the $\alpha$-$\beta$ and *x*-*y* subspaces for a dual three-phase two-level VSC supplying an asymmetrical six-phase IM.

After applying (2), all possible switching states are mapped onto the $\alpha$-$\beta$ and *x*-*y* planes, as shown in Fig. 2. The available voltage vectors can be classified according to their magnitude in the main subspace: large ($V_l$), medium-large ($V_{ml}$), medium ($V_m$), small ($V_s$) and null ($V_0$)^31^. $V_l$ is extensively used in control schemes^32,33^ due to its high $\alpha$–$\beta$ and low *x*–*y* voltage production compared to other options, which enables high achievable torque and flux with low stator copper losses caused by secondary components.

Furthermore, it is useful in terms of control to decouple flux and torque regulation. This can be achieved thanks to the Park transformation, which project the $\alpha$-$\beta$ components onto a rotational reference frame, *d*-*q*:

4 $$\begin{aligned} & [D] = \begin{bmatrix} \cos \theta _s & \sin \theta _s \\ -\sin \theta _s & \cos \theta _s \end{bmatrix} , \end{aligned}$$

5 $$\begin{aligned} & \begin{bmatrix} v_{ds}, v_{qs} \end{bmatrix} ^T = [D] \cdot \begin{bmatrix} v_{\alpha s}, v_{\beta s} \end{bmatrix}^T . \end{aligned}$$

where $\theta _s$ is the angle of the reference frame.

## Dead-time influence estimation

The performance of an electric drive can be influenced by many factors that are commonly considered. Some examples are the voltage drop on the power devices, the time delay in gate drive circuits or the effect that the parasitic capacitance has on the turn-on and turn-off times that switches require^34^. However, the most impactful factor in drive performance is the delay required between the switching of the two semiconductors in the same leg, in order to avoid leg short circuits, also known as dead time^35^. This phenomenon deserves a thorough explanation to better comprehend its negative side effects.

**Fig. 3:**
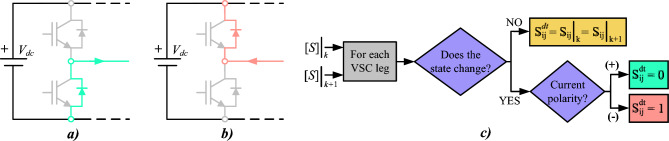
Dead-time effect with different current polarity: (**a**) Positive and (**b**) Negative. (**c**) Flowchart for determining $[S^{dt}]$.

Ideally, the gate control signals of the upper and lower semiconductors on the same phase leg are strictly complementary. Nevertheless, when the switch state changes, the mentioned dead time must be deployed to avoid dc-link short circuit. During this interval, both power switches are open, and the load current flows through one of the freewheeling diodes. Depending on current polarity, two different scenarios appear:

- Positive current polarity (Fig. 3a): Current flows through lower freewheeling diode and pole voltage is clamped to zero volts during dead time.
- Negative current polarity (Fig. 3b): Current flows through upper freewheeling diode and pole voltage is held at $V_{dc}$ volts during dead time.

That is, the appearance of a transitional switching state ($[S^{dt}]$) can generate undesirable voltages in the different subspaces. In fact, to consider the dead-time effect in FCS-MPC, some works have proposed modeling it as a voltage vector that is involuntarily applied^22,35^ (henceforward, dead-time voltage vector $V_{dt}$), resulting from the transition between two VSC switching states. In order to determine $[S^{dt}]$, it is essential to know three key factors: the previously applied switching state $[S]|_k$, the control action to be applied next $[S]|_{k+1}$, and the polarity of the current in the phases whose state changes. The vector $[S^{dt}]$ is defined based on the status of each phase during the dead time, as determined by the procedure described in the flowchart (see Fig. 3c). Table 1 presents an example of the calculation of $[S^{dt}]$ that arises during the switching process between active vectors 9 and 22 (see Fig. 2) for a given current state.

It is demonstrated in^22^ that the effect of $V_{dt}$ degrades the performance of three-phase machines by worsening the $\alpha$-$\beta$ current tracking. The reason behind this fact is that the control scheme is unknowingly applying an undesired voltage vector between the chosen control actions. In multiphase drives, this issue becomes more critical due to the existence of the secondary *x*-*y* plane, usually characterized by a low impedance^36^. This low impedance amplifies the effect of small voltage levels, inducing high current magnitudes that contribute to spoil current quality, increasing power losses in the machine. For that reason, $V_l$ are commonly employed as active control actions because they generate minimal voltage in the *x*-*y* plane^32,33^. However, the example in Table 1 illustrates that switching between two $V_l$ (e.g., vectors 9 and 22) can result in a $V_{dt}$ classified as small in $\alpha$-$\beta$ but large in *x*-*y* (e.g., vector 12), introducing a detrimental effect on the drive efficiency.

**Table 1:** An example of dead-time vector determination.

Phase	$[S_1] = 9$	$[S_2] = 22$	Polarity	$[S^{dt}] = 12$
$a_1$	0	0	Ignored	0
$b_1$	0	1	+	0
$c_1$	1	0	−	1
$a_2$	0	1	−	1
$b_2$	0	1	+	0
$c_2$	1	0	+	0

## MPC for multiphase machines

### Standard MPC

FCS-MPC is based on predicting future drive states using a discretized mathematical model of the machine (hereafter, predictive model), in order to the select the control action that minimizes the deviation from the desired future state. In electric drives, these states are generally the VSD stator currents $(i_{\alpha \beta xys})$, and the control actions correspond to the switching states of the VSC^5^. The standard structure of FCS-MPC typically consists of two different control loops. The outer one uses a proportional-integral (PI) controller to regulate the speed, whereas the inner loop uses the predictive model to regulate the currents. For the electrical drive under consideration, the machine model in space state variables can be represented as follows:

6 $$\begin{aligned} \begin{aligned} \frac{d}{dt} \begin{bmatrix} X_{\alpha \beta xy} \end{bmatrix} =&\begin{bmatrix} A \end{bmatrix} \begin{bmatrix} X_{\alpha \beta xy} \end{bmatrix} + \begin{bmatrix} B \end{bmatrix} \begin{bmatrix} U_{\alpha \beta xy} \end{bmatrix},\\ \begin{bmatrix} U_{\alpha \beta xy} \end{bmatrix} =&\begin{bmatrix} v_{\alpha s} ,\! v_{\beta s} ,\! v_{xs},\! v_{ys}, \!0,\! 0 \end{bmatrix}, \\ \begin{bmatrix} X_{\alpha \beta xy} \end{bmatrix} =&\begin{bmatrix} i_{\alpha s},\! i_{\beta s},\! i_{xs},\! i_{ys},\! \lambda _{\alpha r},\! \lambda _{\beta r} \end{bmatrix}^T, \end{aligned} \end{aligned}$$

where matrices [*A*] and [*B*] describe the dynamic behavior of a six-phase IM and are parameter-dependent. To effectively employ the predictive model, the expression (6) has been discretized through the application of the Euler forward method.

With the objective of meeting real-time requirements, it is necessary to implement the one-step delay (OSD) compensation^37^. This control solution involves evaluating the predictive model twice consecutively to calculate the predicted currents two steps ahead in time $(k+2)$. The total error associated to each switching state current response is quantified by means of a predefined cost function (7), and the control action that minimizes its value is selected.

7 $$\begin{aligned} \begin{aligned} J = e_{\alpha s}^2+e_{\beta s}^2+K_{xy} (e_{xs}^2+e_{ys}^2), \end{aligned} \end{aligned}$$

where:

8 $$\begin{aligned} \begin{aligned} e_{ms} = i^*_{ms}|_{k+2}-\hat{\imath }_{ms}|_{k+2} , \forall m \in \{\alpha , \beta , x , y \} \end{aligned}, \end{aligned}$$

being $K_{xy}$ a weighting factor related to the *x*-*y* currents, and the superscripts “ $\hat{~}$ ” and “ * ” meaning predicted and reference values, respectively. The *q* current reference is determined by the external speed controller, while the *d* current reference is usually set constant to provide the rated stator flux in the base speed region. These reference values are rotated into the stationary $\alpha$-$\beta$ reference frame using the inverse Park transformation. Regarding the *x*-*y* reference currents, they are commonly set to zero, as they are purely related to copper losses in machines with distributed-winding stator configurations.

The switching state selected through the cost function (7) seeks to achieve the control objectives. Unfortunately, applying a single switching state per control period inherently limits the ability to simultaneously satisfy the output voltage requirements in both planes. This limitation can result in undesired harmonic injection in the secondary subspace when the control prioritizes the requirements of the primary plane. To address this problem, various multivector approaches have been proposed in the literature^11^.

### Multivector MPC

FCS-MPC based on multivector control actions has become a hotspot of research in recent years^38,39^. These techniques apply multiple switching states per control period to compose control actions that simultaneously meet the requirements in all subspaces.

**Fig. 4:**
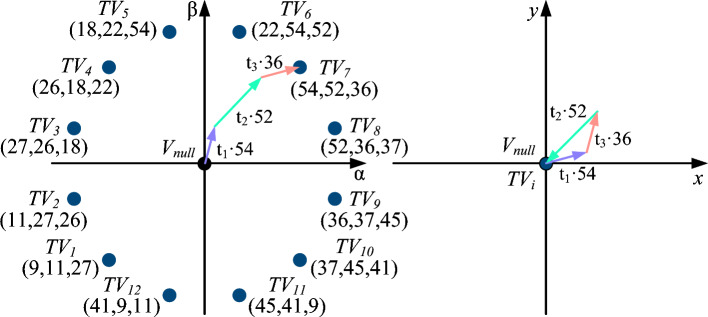
Control action set of TV-MPC neglecting dead-time effect.

In the considered six-phase IM, if the dead-time effect is neglected, control actions with a zero average in the *x*-*y* plane can be achieved through the application of a set of three adjacent large vectors ($V_{l}^1$, $V_{l}^2$ and $V_{l}^3$)^40^. The per-unit duty cycles of each vector is $t_1$= 0.2679, $t_2$=0.4642 and $t_3$=0.2679 respectively, being $V_{l}^2$ located between the two other vectors^40^. Throughout this work, the method utilizing these control actions will be referred to as triple vector model predictive control (TV-MPC). This control strategy employs a modified cost function which only considers $\alpha$-$\beta$ errors since the regulation in the *x*-*y* subspace is performed in open-loop mode. It evaluates thirteen control actions in each sampling period (see Fig. 4) :

- Twelve multivector actions composed of adjacent $V_l$ trios. Henceforth denoted as $TV_i, ~ i \in \{1, 2, \dots , 12\}$.
- One single-vector action that is entirely null in both subspaces, designated as $V_{null}$.

The TV-MPC method will be employed as a benchmark, as it constitutes the simplest multivector control approach that ideally achieves a null average voltage in the secondary subspace by applying only $V_l$. Furthermore, this method will highlight the challenges encountered by multivector control strategies at high frequencies and will provide the foundational basis for the development of the control method proposed in this work.

### Multivector MPC at high frequencies

**Fig. 5:**
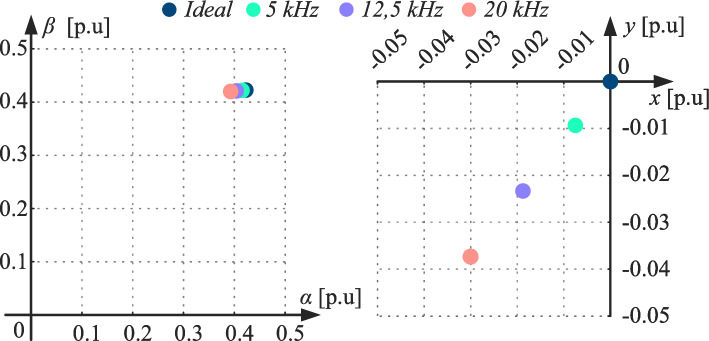
Resulting average voltage of the same TV-MPC control action at different frequencies, considering the dead-time effect.

Although dead time is always present in the VSC, its impact is negligible at low frequencies and can therefore be disregarded. Nonetheless, as the frequency grows, the application time (duty) of each voltage vector decreases while the dead time remains constant, becoming a significant factor. Figure 5 compares the mean voltages obtained in both subspaces applying the virtual vector $TV_7$ at various control frequencies, taking into consideration the impact of the dead-time effect. The comparison includes the ideal case as a reference. Theoretically, the proposed control action averages zero voltage in the secondary subspace. However, the dead-time effect results in a non-zero value, which increases the *x*-*y* currents due to the low impedance of the secondary plane. The voltages, included in Fig. 5, have been calculated for a dead-time duration equal to that of the VSC used on the experimental testing in this work (see *Experimental results* section). As can be observed, the higher the frequency, the greater the deviation of the resulting voltage from the ideally expected value, thereby exacerbating the issue caused by the dead time.

A theoretical study has been carried out to analyze the effect of dead time on the currents in the secondary plane when applying the considered multivector control actions. The *RL* circuit corresponding to the *x*-*y* subspace has been modeled to obtain the behavior of the nonlinear currents:

9 $$\begin{aligned} \begin{aligned} i_{xs} = \frac{v_{xs}}{R_s} + \left( i_{xs0} - \frac{v_{xs}}{R_s} \right) \cdot e^{-t/\tau _s} ,\\ i_{ys} = \frac{v_{ys}}{R_s} + \left( i_{ys0} - \frac{v_{ys}}{R_s} \right) \cdot e^{-t/\tau _s} ,\\ \end{aligned} \end{aligned}$$

where $\tau _s$ is the stator time constant and $R_s$ is the stator resistance. The computation is performed using the parameters of the selected IM, shown in Table 2. The initial currents, denoted as $i_{xs0}$ and $i_{ys0}$, are initialized to zero.

**Fig. 6:**
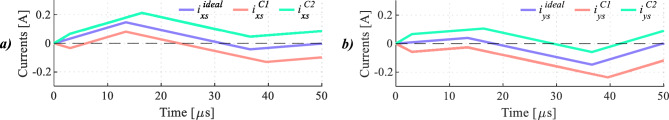
Theoretical evolution of the *x*-*y* currents for the application of the same TV-MPC control action and different conditions. (**a**) *x*-current. (**b**) *y*-current.

Figure 6 shows the evolution of currents in the secondary subspace at a frequency of 20 kHz, under the application of a control action $TV_7$. It compares the ideal case with two scenarios, *C*1 and *C*2, each with distinct conditions, where the polarity of the currents and the initial vector differ. Despite applying the same control action, the effect of dead time on the currents depends on the aforementioned conditions. While the *x*-*y* currents should ideally return to zero at the end of the control period, the presence of dead time introduces an offset in this value (see Fig. 6). Moreover, the instantaneous peak value is also higher when this effect is considered.

Taking into consideration this phenomenon, it extends to reason that an algorithm that accounts for the high variability in secondary currents due to dead time will better exploit the advantages of multivector solutions.

## Proposed dead-time influence estimation MPC

The objective of the dead-time influence estimation model predictive control (DIE-MPC) is to characterize the effect of dead time on multiphase electric drive behavior, allowing the control method to consider its impact. In this vein, the designed predictive algorithm uses the same control actions as TV-MPC but provides the control method with a better understanding of the effects of their application. This additional knowledge enables the selection of optimal actuation, even at high control frequencies.

Neglecting dead time results in a detrimental effect on drive control, which manifests in two distinct aspects:

- In the assessment of the available control actions. As illustrated in Fig. 5, the resulting average voltage in each subspace deviates from the ideal one, thereby leading to an inaccurate evaluation of the control action.
- In the OSD compensation to calculate the future currents in the drive, caused by the control action selected in the previous period. The effect of the action previously chosen as optimal does not align with expectations, resulting in an error in the initial state considered for evaluating future control actions.

**Fig. 7:**
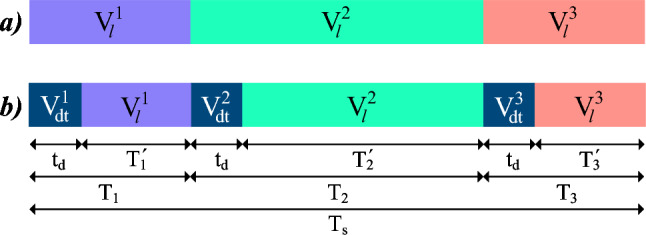
Vectors applied during a TV-MPC control action. (**a**) Ideal case and (**b**) considering dead-time effect.

DIE-MPC addresses the two previously identified issues. To this end, it calculates the resulting average voltage of the control action, taking into account the dead time. Figure 7a depicts the ideal case, where three adjacent $V_l$ are applied, alongside the real case (Fig. 7b), in which three additional $V_{dt}$ are present. In this way, the average voltage in each subspace (henceforward, denoted as $\bar{V}$) can be obtained as follows:

10 $$\begin{aligned} \begin{aligned} \bar{V} = K_{dt} \cdot \sum _{i=1}^{3} V_{dt}^i + \sum _{i=1}^{3} K_i \cdot V_{l}^i, \end{aligned} \end{aligned}$$

where:

11 $$\begin{aligned} \begin{aligned} K_{dt}&= t_d/T_s, \\ K_{i}&= (T_s \cdot t_i - t_d)/T_s, \\ \end{aligned} \end{aligned}$$

with $T_s$ representing the sampling time, $t_d$ the dead time and $t_i$ the ideal per-unit duty cycle of each $V_l$. Moreover, the three $V_{dt}$ are calculated using the procedure described in *Dead-time influence estimation* section.

**Fig. 8:**
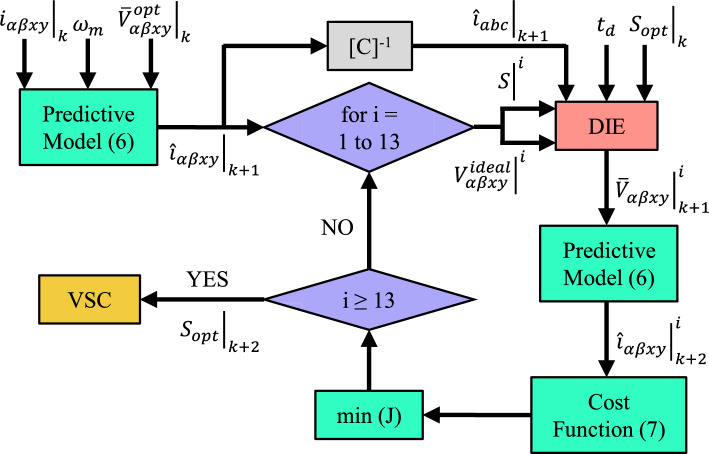
DIE-MPC flowchart.

The flowchart of the proposed control scheme is shown in Fig. 8. Focusing on the proposed strategy, the predictive model is first employed with the previously calculated average voltage $\bar{V} (k)$, accounting for dead time, to determine the phase currents at $k+1$. Next, the 13 available control actions are evaluated using the DIE method, which provides the average voltage $\bar{V} (k+1)$ based on:

- Polarity of the phase currents at $k+1$.
- Switching state applied at *k* and the one being evaluated.
- Ideal voltage provided by the evaluated action.

Subsequently, VSD currents at instant $k+2$ are estimated for each action, and the optimal switching state is selected as the one that minimizes the predefined cost function (7). Despite using multivector actions designed to theoretically average zero in the *x*-*y* plane, the employed cost function considers now the error generated by dead time in both subspaces. This marks a key difference between the proposed method and the ideal virtual vector solutions previously developed.

To sum up, the proposed DIE-MPC achieves a better selection of the optimal action compared to TV-MPC, due to its additional knowledge about dead time. The experimental results presented in the following section confirm the impact of this enhanced multivector FCS-MPC on current quality and other relevant metrics.

## Experimental results

### Test bench

**Fig. 9:**
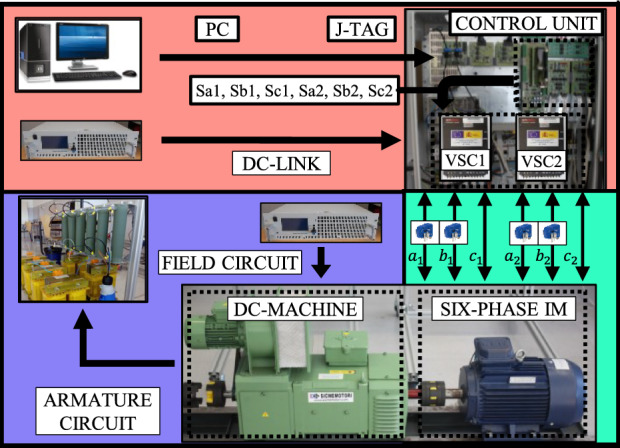
Scheme of the test bench employed to acquire experimental results.

The test bench employed to evaluate the performance of the control schemes is shown in Fig. 9. The six-phase drive consists of an asymmetrical IM supplied from a pair of conventional two-level three-phase VSCs (Semikron SKS22F modules). Additionally, a single dc source is responsible for powering the VSCs. The parameters of the custom-built multiphase IM can be observed in Table 2.

**Table 2:** Six-Phase IM drive and control parameters.

Parameter	Description	Value
$V_{dc}$ (*V*)	dc-link voltage	300
$\omega _{rated}$ (rpm)	Rated speed	1000
$I_{peak}$ (*A*)	Peak current	6.5
$R_s$ ($\Omega$)	Stator resistance	4.19
$R_r$ ($\Omega$)	Rotor resistance	3.2
$L_{m}$ (*mH*)	Mutual inductance	280
$L_{ls}$ (*mH*)	Stator leakage inductance	4.2
$L_{lr}$ (*mH*)	Rotor leakage inductance	55.1
$t_{d}$ ($\mu s$)	VSC dead time	3
$K_{xy}$	*x*-*y* subspace weighting factor	1.5

**Table 3:** Computational burden of control schemes.Computational burden of control schemes.

Method	Computational burden ($\mu s$)
TV-MPC	25
DIE-MPC	39
FCS-MPC	67

Control methods are implemented in a digital signal processor from Texas Instruments (TMS320F28335) programmed using a JTAG and TI proprietary software called Code Composer Studio. Table 3 presents the computational burden of the proposed DIE-MPC, the selected TV-MPC and the conventional FCS-MPC when all available switching states are considered. The sensors that acquire current and speed signals are four hall-effect sensors (LEMLAH25-NP) and a digital encoder (GHM510296R/2500), respectively. The IM is loaded by coupling it to a dc machine by the shaft, thus working as a generator. The armature of the dc machine is connected to a resistive circuit that dissipates power. The load torque is consequently dependent on speed.

### Experimental validation

This section shows experimental results with the purpose of confirming the advantages of the designed DIE-MPC over the previously exposed TV-MPC, which does not consider the effect of dead time. To achieve this objective, harmonic distortion index (HDI)^41^, total harmonic distortion (THD) for the phase $a_1$ based on IEEE Standard 519-22^42^ and switching frequency ($f_{sw}$) have been used as quality indices in the performance evaluation of the multiphase drive under study. Likewise, the product of HDI and $f_{sw}$ is interesting to compare different methods operating under the same conditions^20^.

First, the effect of the defined weighting factor $K_{xy}$ in designed DIE-MPC is evaluated. For that purpose, six different values of this implementation parameter are tested, as shown in Table 4. To analyze the behavior of DIE-MPC, HDI, THD, $f_{sw}$ and the root mean square error (RMSE) of the *d*-*q* currents are added to Table 4. Based on Table 4, when this implementation parameter is not nullified, the method does not exhibit significant sensitivity to variations in the weighting factor. The low dependence on the weighting factor demonstrates the robustness of the method from the perspective of the tuning process. Therefore, a heuristic trial-and-error procedure can be used to tune this parameter with minimal impact on performance^43^.

**Table 4:** Quality indices for different tunings of the $K_{xy}$ weighting factor, $\omega = 500 \, rpm$.

$K_{xy}$	HDI (%)	THD (%)	$f_{sw}$ (kHz)	RMSE_d_ (A)	RMSE_q_ (A)
0	12.76	9.78	7.99	0.06	0.10
1	9.95	6.30	7.79	0.08	0.12
2	9.32	5.01	7.56	0.09	0.12
3	9.31	4.77	7.63	0.11	0.12
4	9.46	4.25	7.61	0.12	0.14
5	9.58	3.74	7.62	0.13	0.14

Secondly, the influence of the control frequency on dead-time effects and the capability of the considered control schemes to mitigate their impact are analyzed. Table 5 presents the values of the mentioned quality indices at the same operating point ($\omega$ = 800 rpm) for four different control frequencies, including the corresponding relative improvement ($\Delta$) for HDI and THD, expressed relative to the TV-MPC results. The results show that the performance of both methods at a low control frequency ($f_c$ = 5 kHz) is similar, since the influence of dead time is not significant. In contrast, for higher control frequency values (12.5, 15.625 and 20 kHz), considering the effect of dead time in the selection of control actions effectively reduces HDI and THD levels without increasing switching losses. This is because the proposed method mitigates the error made in the selection of voltage vectors and ensures a more accurate prediction. For the same dead time ($t_d$ = 3 $\mu$s), the higher the control frequency is, the greater error exists in TV-MPC, and consequently there is greater room for improvement, as shown in $\Delta _{HDI}$ and $\Delta _{THD}$ terms of Table 5. For instance, compared with TV-MPC, the results obtained with DIE-MPC show a notable enhancement in HDI and THD indices, with reductions of 31.70% and 49.93%, respectively, at 20 kHz. Therefore, the proposed method allows for a more effective use of the drive potential at higher control frequencies.

**Table 5:** Quality indices in a range of $f_c$, $\omega = 800 \, rpm$.

$f_c$ (kHz)	Method	HDI (%)	$\Delta _{HDI}$ (%)	THD (%)	$\Delta _{THD}$ (%)	$f_{sw} (kHz)$	HDI $\cdot f_{sw}$
5	TV-MPC	15.36	3.51	2.88	9.72	1.89	29.03
	DIE-MPC	14.82		2.60		1.91	28.30
12.5	TV-MPC	11.31	18.13	4.87	38.40	4.82	54.51
	DIE-MPC	9.26		3.00		4.71	43.61
15.625	TV-MPC	11.62	27.02	6.18	42.23	5.98	69.49
	DIE-MPC	8.48		3.57		5.95	50.46
20	TV-MPC	11.20	31.70	7.03	49.93	7.51	84.13
	DIE-MPC	7.65		3.52		7.44	56.92

**Fig. 10:**
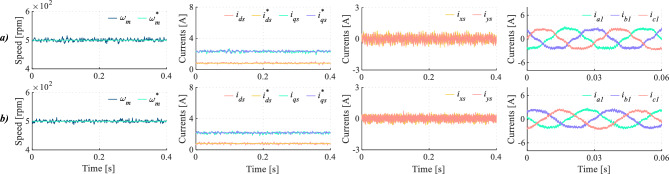
Medium-speed test for (**a**) TV-MPC and (**b**) DIE-MPC. From left to right: Speed, *d*-*q* currents, *x*-*y* currents and phase currents when $f_c$=20 kHz.

**Fig. 11:**
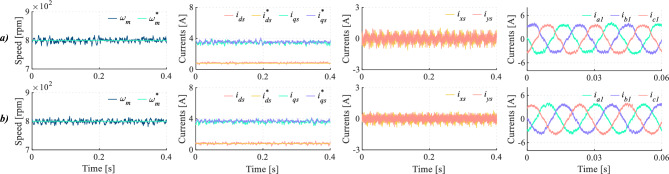
High-speed test for (**a**) TV-MPC and (**b**) DIE-MPC. From left to right: Speed, *d*-*q* currents, *x*-*y* currents and phase currents when $f_c$=20 kHz.

Focusing on high control frequencies, multiple tests have been performed at 20 kHz. The results of these tests are summarized in Table 6. Regardless of the considered case, HDI and THD levels are improved without increasing the switching frequency, resulting in a lower product of HDI and $f_{sw}$ for the proposed method. Furthermore, Figs. 10 and 11 show that, for two different operating points, DIE-MPC reduces currents in the *x*-*y* subspace without compromising the tracking of speed and current references in the *d*-*q* plane, leading to phase currents with lower harmonic distortion. Expanding this analysis, through the application of the fast Fourier transform at $\omega$ = 800 rpm, DIE-MPC achieves a reduction of 53.83% and 31.55% of the 5$^{th}$ and 7$^{th}$ harmonic, reflecting *x*-*y* currents reduction^44^.

**Table 6:** Quality indices in a range of speed, $f_c=20 \,kHz$.

Speed (rpm)	Method	HDI (%)	$\Delta _{HDI}$ (%)	THD (%)	$\Delta _{THD}$ (%)	$f_{sw} (kHz)$	HDI $\cdot f_{sw}$
500	TV-MPC	12.05	23.90	8.96	50.33	8.19	98.69
	DIE-MPC	9.17		4.45		7.71	70.70
650	TV-MPC	11.53	31.92	8.18	52.81	7.91	91.20
	DIE-MPC	7.85		3.86		7.63	59.89
800	TV-MPC	11.20	31.70	7.03	49.93	7.51	84.13
	DIE-MPC	7.65		3.52		7.44	56.92

**Fig. 12:**
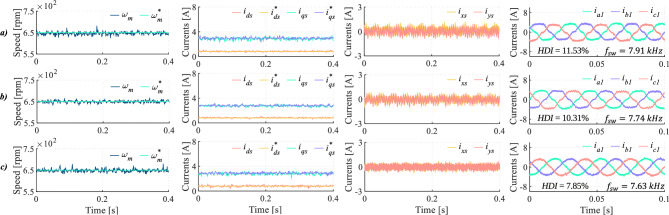
Test at 650 rpm for (**a**) TV-MPC, (**b**) DIE-MPC ($K_{xy}=0$) and (**c**) DIE-MPC ($K_{xy}=1.5$). From left to right: Speed, *d*-*q* currents, *x*-*y* currents and phase currents when $f_c$=20 kHz.

In multiphase drives, mitigating dead-time impact on *x*-*y* currents is essential to ensure high current quality. This requirement becomes even more critical than the influence on the main components, as it occurs in three-phase drives. In order to illustrate this issue, Fig. 12 shows a comparative test between TV-MPC and the proposed method. For the designed DIE-MPC two different configuration solutions have been considered. In the first case Fig. 12b, DIE-MPC has been implemented without considering the control of the secondary currents in the cost function, i.e. using $K_{xy}$ = 0. In the second case Fig. 12c, DIE-MPC has been implemented with $K_{xy}=1.5$. As shown in Fig. 12, taking into account dead-time effect is not sufficient to achieve a significant improvement in terms of HDI in the phase currents if secondary currents are not considered. Fortunately, when *x*-*y* currents are incorporated into the control loop, a proper balance can be established between satisfying magnetic flux and torque requirements and reducing secondary subspace loss currents. This is evidenced by a reduction in the ripple of the *x*-*y* currents and an overall improvement in the quality of phase currents. In conclusion, for multiphase drives the mitigation of dead-time impact on secondary currents is a must.

**Fig. 13:**
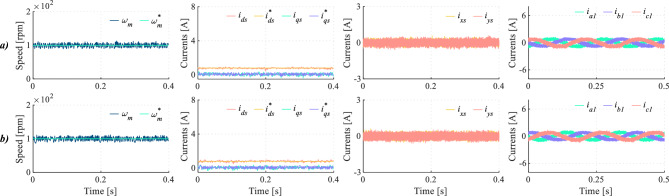
No-load operation at 100 rpm for (**a**) TV-MPC and (**b**) DIE-MPC. From left to right: Speed, *d*-*q* currents, *x*-*y* currents and phase currents when $f_c$=20 kHz.

**Fig. 14:**
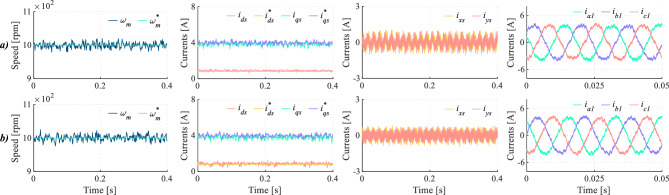
Rated speed test for (**a**) TV-MPC and (**b**) DIE-MPC. From left to right: Speed, *d*-*q* currents, *x*-*y* currents and phase currents when $f_c$=20 kHz.

To provide a comprehensive overview of the DIE-MPC capability to mitigate dead-time effects over a wide range of operating conditions, both low speed ($\omega = 100$ rpm) under no-load torque and rated speed are evaluated. As shown in Fig. 13, under no-load conditions at 100 rpm, the proposed DIE-MPC strategy achieves accurate speed tracking and proper regulation of the *d*-*q* currents. The results summarized in Table 7 indicate that DIE-MPC outperforms the TV-MPC strategy in terms of the evaluated performance indices. This improved performance can be attributed to the explicit consideration of dead-time effects in the multiphase drive under study. In fact, the low-order harmonics associated with dead time are significantly reduced (see Table 7). At the rated motor speed (Fig. 14), the same trend is observed with respect to dead-time mitigation. DIE-MPC achieves lower HDI and THD values, due to the effective attenuation of the 5$^{th}$ and 7$^{th}$ harmonics.

**Table 7:** Quality indices at the analysis boundary with $f_c$=20kHz, where $\dagger$ denotes approximately zero load torque.

Speed (rpm)	Method	HDI (%)	THD (%)	5$^{th}$ harm. (%)	7$^{th}$ harm. (%)
$100^\dagger$	TV-MPC	22.68	6.17	5.08	1.66
	DIE-MPC	22.68	5.06	2.33	0.94
1000	TV-MPC	11.92	6.03	5.44	2.05
	DIE-MPC	8.90	3.69	2.02	0.63

**Fig. 15:**
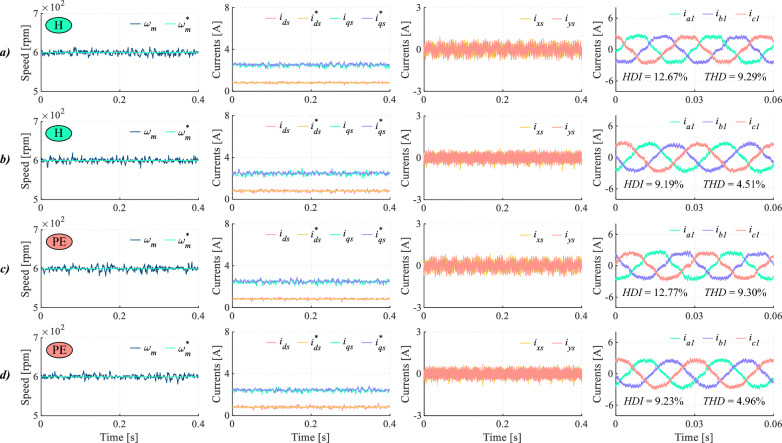
Comparison of results under healthy (H) conditions and model parameter error (PE) for (**a**) TV-MPC (H), (**b**) DIE-MPC (H), (**c**) TV-MPC (PE) and (**d**) DIE-MPC (PE). From left to right: Speed, *d*-*q* currents, *x*-*y* currents and phase currents when $f_c$=20 kHz.

The mitigation of dead-time effects using DIE-MPC under parameter mismatch conditions is also investigated (Fig. 15). In particular, the magnetizing inductance $L_m$ was doubled and the rotor resistance $R_r$ was halved, following the procedure previously used in^45^. As shown in Fig. 15, under parameter mismatch conditions, DIE-MPC achieves lower THD and HDI values than TV-MPC operating under nominal conditions. These results confirm the robust performance of the DIE-MPC strategy regardless of the considered scenario.

**Fig. 16:**
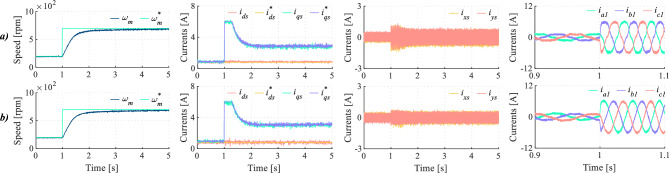
Dynamic test for (**a**) TV-MPC and (**b**) DIE-MPC. From left to right: Speed, *d*-*q* currents, *x*-*y* currents and phase currents when $f_c$=20 kHz.

Finally, Fig. 16 presents a dynamic test evaluating both regulation schemes. This test aims to confirm the effectiveness of the proposed method in mitigating harmonic distortion under varying conditions, preserving the control of the main components. As it can be seen in Figs. 16a and 16b, speed and *d*-*q* currents regulation have an appropriate tracking in both cases. However, *x*-*y* currents are notably lower in DIE-MPC than in TV-MPC (see Fig. 16c), specially when transition state is occurring. This improvement is achieved thanks to the better control actions selection of the considered DIE-MPC. As a consequence of considering dead-time effect on cost function, phase currents show a lower harmonic distortion in this transient scenario, as shown in Fig. 16d.

## Conclusion

The usage of VVs at high control frequencies presents itself as an interesting option to fully exploit the advantages of multiphase systems and FCS-MPC. Nevertheless, this scenario provides new complications, such as the undesirable and now notable effect of the VSC dead time. This phenomenon is translated into an error between the voltage vector modeled in the control algorithm and the real output, which leads to poorer control action selection and worse overall performance. This work tackles this issue, providing a theoretical analysis of the problem in order to bring further insight into the consequences of using multivector MPC strategies at increasing values of the control frequency. Moreover, the model of an FCS-MPC scheme based on multivector actions is improved by including knowledge of the dead-time effect. The experimental results of the proposed DIE-MPC back the importance of considering this phenomenon, providing a reduction of both the switching frequency and the current harmonic content at different operating points and control frequencies. In fact, this improvement in current quality is also achieved under dynamic conditions without affecting the transient response of the electric drive.

## Data Availability

The datasets generated and/or analysed during the current study are available in the RIUMA repository, [https://hdl.handle.net/10630/40581].
